# Prognostic Value of the AST/ALT Ratio versus Bilirubin in Patients with Cardiogenic Shock

**DOI:** 10.3390/jcm12165275

**Published:** 2023-08-14

**Authors:** Tobias Schupp, Jonas Rusnak, Kathrin Weidner, Marinela Ruka, Sascha Egner-Walter, Jonas Dudda, Jan Forner, Thomas Bertsch, Kambis Mashayekhi, Mohamed Ayoub, Muharrem Akin, Maximilian Kittel, Michael Behnes, Ibrahim Akin

**Affiliations:** 1 Department of Cardiology, Angiology, Haemostaseology and Medical Intensive Care, University Medical Centre Mannheim, Medical Faculty Mannheim, Heidelberg University, 68167 Mannheim, Germany; tobias.schupp@umm.com (T.S.);; 2 European Center for AngioScience (ECAS), German Center for Cardiovascular Research (DZHK) Partner Site Heidelberg/Mannheim, 68167 Mannheim, Germany; 3 Institute of Clinical Chemistry, Laboratory Medicine and Transfusion Medicine, Nuremberg General Hospital, Paracelsus Medical University, 90419 Nuremberg, Germany; 4 Department of Internal Medicine and Cardiology, Mediclin Heart Centre Lahr, 77933 Lahr, Germany; 5 Division of Cardiology and Angiology, Heart Center University of Bochum, 32545 Bad Oeynhausen, Germany; 6 Department of Cardiology and Angiology, Hannover Medical School, 30625 Hannover, Germany; 7 Institute for Clinical Chemistry, Faculty of Medicine Mannheim, Heidelberg University, 68167 Mannheim, Germany

**Keywords:** cardiogenic shock, AST/ALT ratio, bilirubin, biomarkers, prognosis, mortality

## Abstract

This study investigates the prognostic value of the aspartate-to-alanine aminotransferase ratio (i.e., AST/ALT ratio) and bilirubin in patients with cardiogenic shock (CS). Despite ongoing improvements regarding the treatment of CS patients, invasive care unit (ICU) mortality in CS patients remains unacceptably high. Limited data regarding the prognostic value of the AST/ALT ratio and bilirubin in patients suffering from CS is available. The authors hypothesize the measurement of liver enzymes during the course of CS may be an easy and feasible method to assess right-heart dysfunction and prognosis in patients with CS. Consecutive patients with CS from 2019 to 2021 were included. Blood samples were retrieved from the day of disease onset (day 1), days 2, 3, 4 and 8. The prognostic value of the AST/ALT ratio and bilirubin was tested for 30-day all-cause mortality. Statistical analyses included univariable *t*-tests, Spearman’s correlations, Kaplan–Meier analyses, as well as multivariable Cox proportional regression analyses. A total of 157 CS patients were included, with an overall rate of all-cause mortality at 30 days of 51%. The median AST/ALT ratio on day 1 was 1.4, and the median bilirubin was 0.63 mg/dL. No association of the baseline AST/ALT ratio (HR = 1.005; 95% CI 0.649–1.558; *p* = 0.981) and bilirubin (HR = 1.320; 95% CI 0.834–2.090; *p* = 0.236) with the risk of 30-day all-cause mortality was found. In contrast, the AST/ALT ratio on day 4 was associated with the risk of 30-day all-cause mortality (HR = 2.826; 95% CI 1.227–6.510; *p* = 0.015), which was still evident after the multivariable adjustment (HR = 2.830; 95% CI 1.054–7.690; *p* = 0.039). The AST/ALT ratio during the course of ICU hospitalization from day 4—but not the baseline AST/ALT ratio and bilirubin—was associated with an increased risk of 30-day all-cause mortality in CS patients.

## 1. Introduction

Due to improved treatment strategies for acute myocardial infarction (AMI), including shorter door-to-balloon times, improved revascularization strategies, nationwide healthcare supply and cardiovascular pharmacotherapies, mortality rates following AMI have significantly improved during the past decades [1,2]. In line with this, the incidence of AMI-related cardiogenic shock (CS) was shown to decrease, whereas the incidence of non-AMI-related CS was shown to increase [3,4]. CS is characterized by low cardiac output, which leads to tissue hypoperfusion and hypoxia [5]. Treatment of CS consists of early revascularization in AMI-related CS, supply with inotropes and catecholamines, as well as the insertion of mechanical circulatory support (MCS) devices [6]. However, despite optimal treatment of CS, in-hospital mortality has remained high, reaching 40–50% [5]. For better risk stratification in CS, a few biomarkers (including creatinine, lactate and glucose) were identified as affecting short-term prognosis [7].

CS may lead to liver dysfunction in heart failure and CS as a result of both tissue hypoperfusion and congestion related to volume and pressure overload [8]. Hepatic transaminases, such as aspartate aminotransferase (AST) and alanine aminotransferase (ALT), as well as the AST/ALT ratio (the so-called “De Ritis” ratio), were shown to be associated with all-cause mortality in various clinical settings, such as patients with cancer, arterial hypertension, pulmonary embolism and sepsis [9,10,11,12,13,14]. Studies investigating the prognostic role of the AST/ALT ratio in patients with predominantly heart failure included patients with chronic heart failure or patients with acute decompensated heart failure in the absence of CS [15,16]. However, heart failure-related liver injury may presumably occur in CS patients with persistent arterial hypotension [8]. In line with this, bilirubin was shown to be an independent predictor of cardiovascular death and a worsening of heart failure [17], whereas the relationship between bilirubin and the outcomes in CS patients remains unclear.

The prognostic value of liver enzymes on the prognosis of patients with CS was investigated in studies predominantly including patients undergoing MCS insertion, with conflicting findings. In 80 patients undergoing veno-arterial extracorporeal membrane oxygenation (VA-ECMO), it was demonstrated that specifically, the normalization of liver enzymes was shown to occur within five days of ICU treatment and elevated liver enzymes were not associated with the risk of all-cause mortality [18]. In contrast, an early increase in bilirubin levels (i.e., after 2 h of MCS insertion) was observed in non-survivors compared to survivors within 103 patients treated with extracorporeal life support (ECLS), whereas other liver enzymes, such as AST, did not differ [19]. In line with this, the “RESCUE” score, including bilirubin, was developed to predict the early risk of mortality in patients treated with ECLS [20]. In contrast, a comprehensive analysis of the AST/ALT ratio and bilirubin is not yet available.

In patients with CS, hepatic congestion may be closely related to right ventricular dysfunction (RVD). RVD, assessed by the tricuspid annular plane systolic excursion (TAPSE), was recently demonstrated to be an independent predictor of all-cause mortality in patients with CS [21]. However, during routine clinical care, echocardiography is infrequently performed during the course of intensive care unit (ICU) hospitalization in patients treated for CS; even within randomized controlled trials (RCT), dynamic changes in right ventricular function are not assessed [22]. 

The measurement of blood-derived biomarkers, such as the AST/ALT ratio or bilirubin, might be a feasible and easily applicable method to detect RVD and its associated prognosis in patients with CS. The authors hypothesize that an elevated and, specifically, an increase of the AST/ALT ratio may indicate an adverse prognosis in patients suffering from CS. Therefore, the present study comprehensively investigates the prognostic role of the AST/ALT ratio and bilirubin, as well as their course during ICU hospitalization in consecutive patients admitted with CS.

## 2. Materials and Methods

### 2.1. Study Patients, Design and Data Collection 

The present study prospectively included all consecutive patients presenting with CS on admission to the internistic ICU at the University Medical Center Mannheim, Germany, from June 2019 to May 2021, as recently published [23]. All relevant clinical data related to the index event were documented using the electronic hospital information system as well as the IntelliSpace Critical Care and anesthesia information system (ICCA, Philips, Philips GmbH Market DACH, Hamburg, Germany) implemented at the ICU, organizing patient data, such as admission documents, vital signs, laboratory values, treatment data and consult notes. 

Important laboratory data, ICU-related scores, hemodynamic measurements and ventilation parameters were assessed on the day of admission (i.e., day 1), as well as on days 2, 3, 4 and 8. Furthermore, the baseline characteristics, prior medical history, length of index hospital stay, data derived from imaging diagnostics, as well as pharmacological therapies were documented. Echocardiographic measurements were performed on day 1 of CS during routine clinical care. Documentation of the source data was performed by intensivists and ICU nurses during routine clinical care. 

This present study was derived from an analysis of the “Cardiogenic Shock Registry Mannheim” (CARESMA-registry) and represents a prospective single-center registry that includes consecutive patients presenting with cardiogenic shock being acutely admitted to the ICU for internal medicine at the University Medical Center Mannheim (UMM), Germany (clinicaltrials.gov identifier: NCT05575856). The registry was carried out according to the principles of the Declaration of Helsinki and was approved by the medical ethics committee II of the Medical Faculty Mannheim, University of Heidelberg, Germany.

The medical center covers a general emergency department (ED) for emergency admission of traumatic, surgical, neurological and cardiovascular conditions. Interdisciplinary consultation is an inbuilt feature of this 24/7 service and connects to a stroke unit, four ICUs and a chest pain unit (CPU) to alleviate the rapid triage of patients. The cardiologic department itself includes a 24 h catheterization laboratory, an electrophysiologic laboratory, a hybrid operating room and telemetry units. Since 2020, the University Medical Center has been a certified cardiac arrest center (CAC), including the ability to implant extracorporeal life support (ECLS) devices, such as Impella and VA-ECMO (i.e., i-cor^®^, Xenios AG, Heilbronn, Germany and Cardiohelp, Getinge, Gothenburg, Sweden) [24,25,26].

### 2.2. Inclusion and Exclusion Criteria, Study Endpoints

For the present study, all consecutive patients with CS and a measurement of the AST/ALT ratio on day 1 were included. Patients that were <18 years of age were excluded. No further exclusion criteria were applied. The diagnosis of CS was determined according to the current recommendations of the Acute Cardiovascular Care Association of the European Society of Cardiology [27,28]. Accordingly, cardiogenic shock was defined by hypotension (SBP < 90 mmHg) for more than 30 min, despite adequate filling status or the need for vasopressor or inotropic therapy to achieve a SBP of >90 mmHg. Additionally, signs of end-organ hypoperfusion must be present, such as oliguria with a urine output of <30 mL/h, altered mental status, cold, clammy skin and an increased lactate of >2 mmol/L. 

All-cause mortality at 30 days was documented using the electronic hospital information system and by directly contacting the state resident registration offices (‘Bureau of Mortality Statistics’). The identification of patients was verified by their place of name, surname, day of birth, and registered living address. No patient was lost to follow-up with regard to all-cause mortality at 30 days. 

### 2.3. Statistical Methods

Quantitative data are presented as the mean ± standard error of the mean (SEM), median and interquartile range (IQR), and ranges depending on the distribution of the data. They were compared using the Student’s *t*-test for normally distributed data or the Mann–Whitney U test for nonparametric data. Deviations from a Gaussian distribution were tested by the Kolmogorov–Smirnov test. Qualitative data are presented as absolute and relative frequencies and were compared using the Chi-square test or Fisher’s exact test, as appropriate. Box plots for the AST/ALT ratio and bilirubin were created for the comparisons of survivors and non-survivors on days 1, 2, 3, 4 and 8. Spearman’s rank correlation for nonparametric data was used to test for the association of the AST/ALT ratio and bilirubin with medical and laboratory parameters on day 1 and on day 4.

### 2.4. Prognostic Performance of the AST/ALT Ratio and Bilirubin

C-statistics were applied by calculating the receiver operating characteristic (ROC) curves and investigating the corresponding areas under the curves (AUC) within the entire cohort in order to assess the diagnostic performance of the AST/ALT ratio and bilirubin during the first week of ICU hospitalization with regard to the 30-day all-cause mortality. Kaplan–Meier analyses, according to the AST/ALT ratio and bilirubin on day 1 and day 4 were performed, and univariable hazard ratios (HR) were obtained together with the 95% confidence intervals. Multivariable Cox regression models were developed using the “forward selection” option.

The results of all statistical tests were considered significant for *p* ≤ 0.05. SPSS (Version 28, IBM, Armonk, New York, NY, USA) and GraphPad Prism (Version 9, GraphPad Software, San Diego, CA, USA) were used for the statistics. 

## 3. Results

### 3.1. Study Population

From 2019 to 2021, 273 patients with CS were admitted to our institution. A total of 116 patients with no AST/ALT ratio on day 1 were excluded. The final study cohort comprised 157 CS patients with a median AST/ALT ratio of 1.40 (IQR 1.04–1.96). Patients were median-aged at 74 years, and most patients were male (62%) (Table 1; left panel). Despite a lower body temperature on admission (35.6 °C vs. 36.3 °C; *p* = 0.006), the vital signs on admission, including the heart rate (86 beats per minute (bpm) vs. 88 bpm; *p* = 0.829) and SBP (110 mmHg vs. 109 mmHg; *p* = 0.927) did not differ among 30-day non-survivors and survivors (Table 1; middle and right panel). Accordingly, the rates of coronary artery disease (39% vs. 36%; *p* = 0.789), atrial fibrillation (36% vs. 40%; *p* = 0.605) and congestive heart failure (40% vs. 39%; *p* = 0.894) were comparable. No differences regarding pre-treatment with pharmacotherapies were observed when comparing non-survivors and survivors. Table 2 illustrates the CS-related data and procedures during ICU hospitalization. AMI was the most common cause of CS but occurred more often in 30-day non-survivors compared to survivors (58% vs. 39%), followed by acute decompensated heart failure (30% vs. 26%) (*p* = 0.008). In line, a LVEF < 30% was most common in the non-survivor group (63% vs. 38%; *p* = 0.021), alongside with higher rates of out-of-hospital (46% vs. 26%; *p* = 0.001) and in-hospital cardiac arrest (21% vs. 7%; *p* = 0.001). Furthermore, higher norepinephrine doses on admission (0.2 µg/kg/min vs. 0.1 µg/kg/min; *p* = 0.001) and higher rates of MCS insertion (19% vs. 3%; *p* = 0.001) were observed in the non-survivors. In contrast, right ventricular function, as reflected by the tricuspid annular plane systolic excursion (TAPSE), did not differ in both groups (median 15 mm vs. 17 mm; *p* = 0.300). With regard to the laboratory data on day 1, especially the lactate levels (4.6 mmol/L vs. 2.6 mmol/L; *p* = 0.001), the white blood cell count (WBC) (15.7 × 10^6^/mL vs. 13.3 × 10^6^/mL; *p* = 0.003), international normalized ratio (INR) (1.2 vs. 1.1; *p* = 0.023) and cardiac troponin I (cTNI) levels (2.86 µg/L vs. 0.46 µg/L; *p* = 0.002) were higher in the non-survivors as compared to the survivors on day 1. In contrast, the AST/ALT levels on admission (1.41 vs. 1.40; *p* = 0.944) and bilirubin (0.65 vs. 0.60 mg/dL; *p* = 0.395) did not differ in both groups (Table 2).

### 3.2. Association of AST/ALT Ratio and Bilirubin with Clinical and Laboratory Data

Table 3 illustrates the correlation of the AST/ALT ratio and bilirubin on day 1 with clinical and laboratory data. On day 1, the AST/ALT ratio correlated with cTNI (r = 0.366; *p* = 0.001), whereas no further correlations with the clinical data and biomarkers were observed. In contrast, bilirubin on day 1 correlated with the C-reactive protein (r = 0.276; *p* = 0.001) and N-terminal pro-B-type natriuretic peptide (NT-pro BNP) levels (r = 0.338; *p* = 0.003), whereas an inverse correlation with the platelet count (r = −0.254; *p* = 0.002) was shown. On the contrary, no correlation between liver enzymes and TAPSE on day 1 was demonstrated. Further correlations during the course of CS are presented in Supplementary Table S1.

### 3.3. Prognostic Performance of the AST/ALT Ratio and Bilirubin

**Figure 1:**
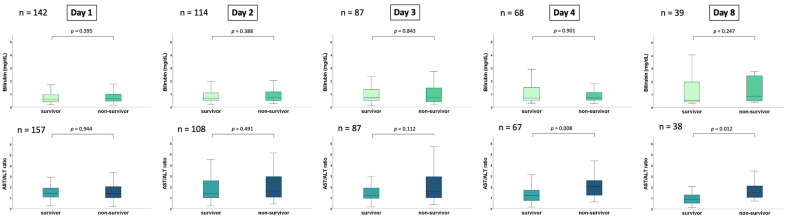
Box plots demonstrate the distribution of the AST/ALT ratio and bilirubin among patients with CS during the first 8 days of CS onset (i.e., 1, 2, 3, 4 and 8). The data are presented as the median with interquartile ranges (boxes) and 5–95% percentiles (whiskers).

Overall, the risk of 30-day all-cause mortality was 51%. During the first week of ICU hospitalization, the bilirubin levels did not differ among 30-day non-survivors as compared to survivors on day 1 (0.65 vs. 0.60 mg/dL; *p* = 0.395), day 2 (0.74 vs. 0.64 mg/dL; *p* = 0.388), day 3 (0.74 vs. 0.71 mg/dL; *p* = 0.843), day 4 (0.70 vs. 0.69 mg/dL; *p* = 0.901) and day 8 (0.87 vs. 0.55 mg/dL; *p* = 0.247) (Figure 1). In line, the AST/ALT ratio did not differ in the non-survivor or survivor groups on day 1 (1.41 vs. 1.40; *p* = 0.944), day 2 (1.61 vs. 1.38; *p* = 0.491) and day 3(1.56 vs. 1.21; *p* = 0.112), whereas the AST/ALT ratio was significantly higher in 30-day non-survivors on day 4 (2.03 vs. 1.20; *p* = 0.008) and day 8 (1.51 vs. 0.89; *p* = 0.012).

The prognostic AUCs of the AST/ALT ratio were not statistically significant from day 1 to day 3 to predict all-cause mortality at 30 days (range of AUC, 0.497 to 0.601); however, they were significant in predicting the risk of 30-day all-cause mortality thereafter on day 4 and day 8 (range of AUC, 0.692 to 0.756). Of note, the prognostic AUCs for bilirubin were not as consistent as those of the AST/ALT ratio on the evaluated treatment days (range of AUC, 0.487 to 0.617) (Table 4).

**Figure 2:**
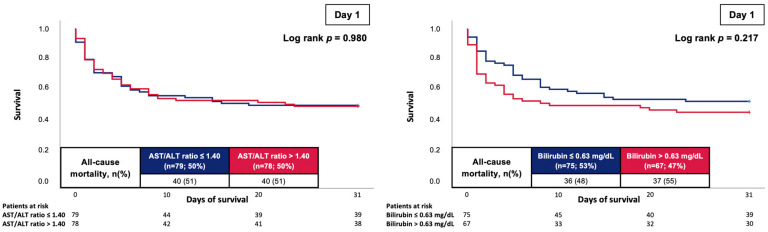
Prognostic impact of the AST/ALT ratio (**left panel**) and bilirubin (**right panel**) on day 1 on the risk of all-cause mortality at 30 days.

At 30 days, the primary endpoint of all-cause mortality occurred in 51% of the patients with an AST/ALT ratio of ≤1.40 (median on day 1) and in 51% of the patients with an AST/ALT ratio of >1.40. Accordingly, the risk of all-cause mortality was not affected by the baseline AST/ALT ratio (log rank *p* = 0.980; HR = 1.005; 95% CI 0.649–1.558; *p* = 0.981) (Figure 2, left panel). In line, no association of the baseline bilirubin with the risk of 30-day all-cause mortality was shown (55% vs. 48%; log rank *p* = 0.217; HR = 1.320; 95% CI 0.834–2.090; *p* = 0.236) (Figure 2, right panel).

**Figure 3:**
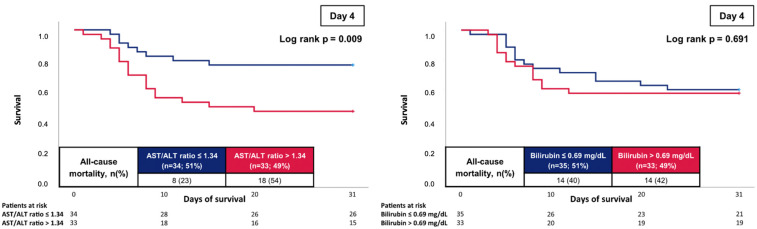
Prognostic impact of the AST/ALT ratio (**left panel**) and bilirubin (**right panel**) on day 4 on the risk of all-cause mortality at 30 days.

However, on day 4, an AST/ALT ratio above the median (i.e., >1.34) was associated with an increased risk of 30-day all-cause mortality as compared to patients with lower values (54% vs. 23%; log rank *p* = 0.009; HR = 2.826; 95% CI 1.227–6.510; *p* = 0.015) (Figure 3, left panel). A higher AST/ALT ratio specifically discriminated the risk of all-cause mortality in patients with a TAPSE of <18 mm (HR = 8.338; 95% CI 1.023–67.972; *p* = 0.048), whereas no association of the AST/ALT ratio in patients with a higher TAPSE was seen. 

In contrast, bilirubin on day 4 was not associated with 30-day mortality (Figure 3, right panel). Furthermore, bilirubin had no prognostic impact in patients with a TAPSE of <18 mm (HR = 1.019; 95% CI 0.294–3.525; *p* = 0.977) and in patients with a normal TAPSE (HR = 2.399; 95% CI 0.329–17.478; *p* = 0.388).

After multivariable adjustment, the AST/ALT ratio on day 4 was still associated with the risk of 30-day all-cause mortality (HR = 2.830; 95% CI 1.054–7.690; *p* = 0.039), whereas bilirubin was not associated with mortality (Table 5; right panel). Furthermore, especially higher lactate levels predicted the risk of 30-day all-cause mortality.

## 4. Discussion

The present study comprehensively investigates the prognostic impact of the AST/ALT ratio and bilirubin in patients admitted with CS. The data suggest no impact of the baseline AST/ALT ratio and bilirubin on the risk of 30-day all-cause mortality in CS patients. However, during the course of ICU hospitalization, the AST/ALT ratio revealed reliable prognostic accuracy for the prediction of 30-day mortality on day 4 and day 8, which was superior to bilirubin. An AST/ALT ratio above the median on day 4 was associated with a significantly increased risk of 30-day all-cause mortality, which was still evident after multivariable adjustment. Specifically, in patients with a TAPSE of < 18 mm, the AST/ALT ratio discriminated the risk of 30-day all-cause mortality and may, therefore, be a reliable predictor to reflect right-heart function among patients with CS. In contrast, bilirubin was not associated with the risk of all-cause mortality during the course of ICU hospitalization.

Abnormal liver function tests represent a common finding in patients with heart failure. In patients with heart failure—and specifically, patients with CS—liver dysfunction may occur as a consequence of increased right-heart cardiac pressure, low-output related impaired perfusion, or is secondary to drug toxicity [8]. However, studies investigating the prognostic impact of liver function tests on patients with heart failure predominantly included patients with chronic heart failure with heterogenous findings, whereas no further risk stratification was performed according to the presence of CS [16]. A higher risk of all-cause mortality in elderly patients with an AST/ALT ratio of ≥1.7 was observed in 1327 patients hospitalized for heart failure who were at least 65 years of age, alongside a higher rate of frailty and malnutrition. However, no parameters of right-heart failure were assessed, and CS was beyond the scope of this study [29]. On the contrary, both AST and ALT were not associated with an impaired prognosis among 1657 patients with heart failure with preserved ejection fraction, which may be related to sufficient remaining cardiac output in those patients [16].

Pathophysiologically, cardiac output may not be sufficient to meet the metabolic demand of hepatic cells in CS [30]. Seeto et al. investigated the association of right-heart failure with AST and ALT levels. Including 31 patients with ischemic hepatitis (94% due to right-heart failure) with concomitant arterial hypotension for at least 15 min, the authors found a significant increase in the AST and ALT levels but not in the controls with hypotension due to non-hepatic trauma, suggesting that hepatic venous congestion may predominantly contribute to ischemic hepatitis [31]. In line with this, higher central venous pressure alongside lower hepatic blood flow was seen in 20 patients suffering from ischemic hepatitis, as compared to patients without [32]. Acute cardiogenic liver injury (ACLI) was demonstrated to result in an increase in the aminotransferases, bilirubin, lactate dehydrogenase (LDH) levels and prothrombin time, which typically occurs with a latency of 1 to 3 days in the absence of another etiology [8,33]. 

However, there is limited data available when focusing on the prognostic value of ACLI in patients with acute heart failure or CS. For instance, a sub-study of the “SURVIVE” (Survival of Patients With Acute Heart Failure in Need of Intravenous Inotropic Support) trial demonstrated an increased risk of 31- and 180-day mortality among 1134 patients with decompensated heart failure and a LVEF of <30% requiring inotropic support. However, all patients required a SBP of > 85 mmHg per inclusion criteria and patients with concomitant CS were excluded by study protocol [34]. In line with these findings, Matthews et al. suggested elevated AST levels were useful in predicting right-heart failure among 197 left ventricular assist device (LVAD) candidates [35]. Furthermore, few studies investigated the prognostic role of liver enzymes in patients with CS treated with MCS. The prognostic impact of the AST, ALT and total bilirubin was retrospectively investigated in 152 patients undergoing ECMO insertion for various reasons (i.e., cardiac or pulmonary failure), suggesting that total bilirubin was associated with the risk of in-hospital mortality after a multivariable adjustment [36]. Similar findings were made by Huang et al., suggesting the total bilirubin peak was associated with the risk of 28-day all-cause mortality in 60 patients with ECMO treatment [37]. In line with this, Tariza et al. investigated the prognostic role of a biomarker-based score for improved risk prediction in patients undergoing MCS insertion for ECLS. Their “RESCUE” score was created using platelet count creatinine and bilirubin to predict the risk of mortality, including 208 patients, taking into account the worst laboratory value within the first nine days of admission, revealing reliable discrimination of all-cause mortality in patients undergoing ECLS (corresponding AUC 0.763) [20]. However, dynamic changes in liver enzymes were not taken into account in these studies. In the setting of CS, dynamic changes in liver enzymes may be in line with hepatic congestion and strongly associated with RVD [21]. From this perspective, the measurement of liver enzymes may be helpful for further risk stratification in CS, which may be related to the easy feasibility and implementation into daily practice. For instance, specific echocardiographic assessment is typically not performed during the course of CS. Even within current and ongoing RCT, echocardiographic assessment is typically performed on CS on day 1, whereas dynamic changes in left and right ventricular function are typically not performed [22].

Of note, the concomitant incidence of infection was 54% in CS patients within the present study. In patients with sepsis, hypoxic hepatitis is a common finding as a consequence of oxygen demand and supply mismatch, aggravated by lipopolysaccharides (LPS) and inflammatory cytokines [38,39,40,41]. In line, an increased AST/ALT ratio was shown to increase the risk of 180-day all-cause mortality using uni- and multivariable Cox regression analyses among 183 septic patients [13].

Finally, pharmacological therapies may contribute to hepatic injury in patients with heart failure or CS by multiple mechanisms, such as acute hepatocellular injury, cholestasis or autoimmune hepatitis [8]. Especially, despite the rather high prevalence of AMI-related CS (48% within the present registry), treatment with clopidogrel and ASA may increase the risk of ACLI as compared to heart failure patients without concomitant CS [8].

This study has several limitations. Due to the single-center and observational study design, the results may be influenced by measured and unmeasured confounding. Patients with out-of-hospital cardiac arrest not transferred to our institution and patients dying before the first AST/ALT ratio and bilirubin measurements could not be included in the study. For the present study, echocardiographic assessment, especially measurements of right ventricular function (such as the TAPSE), was routinely performed on day 1 of ICU treatment but not re-assessed during the course of CS treatment thereafter. Furthermore, the duration of heart failure treatment prior to CS onset was beyond the scope of the present study. Finally, no information on long-term mortality was available for the present study.

In conclusion, liver enzymes on admission were not associated with the risk of 30-day all-cause mortality in consecutive CS patients, whereas an increased AST/ALT ratio on day 4 and beyond was associated with an increased risk of 30-day all-cause mortality. Consistency was shown using multivariable Cox regression analyses. In contrast, bilirubin was not associated with the risk of all-cause mortality.

## Figures and Tables

**Table 1:** Baseline characteristics.

	All Patients (*n* = 157)		Survivor (*n* = 77)		Non-Survivor (*n* = 80)		*p*-Value
**Age**, median (IQR)	74	(64–81)	73	(62–81)	75	(65–81)	0.375
**Male sex**, *n* (%)	98	(62.4)	49	(63.6)	49	(61.3)	0.758
**Body mass index**, kg/m^2^ (median (IQR))	26.60	(24.50–30.40)	26.60	(24.20–29.90)	26.85	(24.70–30.80)	0.453
**Vital signs on admission** (median (IQR))							
Body temperature (°C)	35.9	(35.9–36.6)	36.3	(35.4–36.6)	35.6	(34.5–36.3)	**0.006**
Heart rate (bpm)	87	(71–108)	88	(72–109)	86	(70–107)	0.829
Systolic blood pressure (mmHg)	110	(92–133)	109	(92–130)	110	(93–135)	0.927
Respiratory rate (breaths/min)	20	(18–24)	20	(18–23)	20	(18–24)	0.715
Prior TAPSE, mm (median (IQR))	19	(15–24)	21	(18–25)	16	(13–22)	**0.029**
**Cardiovascular risk factors**, *n* (%)							
Arterial hypertension	118	(75.2)	60	(77.9)	58	(72.5)	0.432
Diabetes mellitus	64	(40.8)	30	(39.0)	34	(42.5)	0.652
Hyperlipidemia	90	(57.3)	47	(61.0)	43	(53.8)	0.356
Smoking	56	(35.9)	31	(40.8)	25	(31.3)	0.214
**Prior medical history**, *n* (%)							
Coronary artery disease	59	(37.2)	29	(36.2)	30	(39.0)	0.789
Congestive heart failure	62	(39.5)	30	(39.0)	32	(40.0)	0.894
Atrial fibrillation	60	(38.2)	31	(40.3)	29	(36.3)	0.605
Chronic kidney disease	61	(38.9)	31	(40.3)	30	(37.5)	0.723
Stroke	22	(14.0)	12	(15.6)	10	(12.5)	0.578
COPD	26	(16.6)	12	(15.6)	14	(17.5)	0.747
Liver cirrhosis	6	(3.8)	5	(6.59	1	(1.3)	0.090
**Prior medical treatment**, *n* (%)							
ACE-inhibitor	49	(34.3)	25	(32.9)	24	(35.8)	0.713
ARB	26	(18.1)	16	(20.8)	10	(14.9)	0.362
Beta-blocker	81	(56.6)	44	(57.9)	37	(55.2)	0.748
ARNI	4	(2.8)	3	(3.9)	1	(1.5)	0.367
Aldosterone antagonist	27	(18.9)	11	(14.5)	16	(23.9)	0.151
Diuretics	76	(52.8)	37	(48.7)	39	(57.4)	0.298
ASA	36	(22.9)	22	(28.6)	14	(17.5)	0.099
P2Y12-inhibitor	12	(7.6)	6	(7.8)	6	(7.5)	0.945
Statin	64	(44.5)	39	(51.3)	25	(36.8)	0.079
Amiodarone	10	(6.4)	4	(5.2)	6	(7.5)	0.554

ACE, angiotensin converting enzyme; ARB, angiotensin receptor blocker; ARNI, angiotensin receptor-neprilysin inhibitor; ASA, acetylsalicylic acid; COPD, chronic obstructive pulmonary disease; IQR, interquartile range; TAPSE, tricuspid annular plane systolic excursion. Level of significance *p* < 0.05. Bold type indicates statistical significance.

**Table 2:** Shock-related data, follow-up data and endpoints.

	All Patients (*n* = 157)		Survivor (*n* = 77)		Non-Survivor (*n* = 80)		*p*-Value
**Cause of CS**, *n* (%)							
Acute myocardial infarction	76	(48.4)	30	(39.0)	46	(57.5)	
Arrhythmic	17	(10.8)	14	(18.2)	3	(3.8)	
Acute decompensated heart failure	44	(28.0)	20	(26.0)	24	(30.0)	
Pulmonary embolism	6	(3.8)	2	(2.6)	4	(5.0)	**0.008**
Vitium	9	(5.7)	6	(7.8)	3	(3.8)	
Cardiomyopathy	2	(1.3)	2	(2.6)	0	(0.0)	
Pericardial tamponade	3	(1.9)	3	(3.9)	0	(0.0)	
**Coronary angiography**, *n* (%)							
No evidence of CAD	16	(10.2)	10	(13.0)	6	(7.5)	0.256
1-vessel-disease	24	(15.3)	12	(15.6)	12	(15.0)	0.919
2-vessel-disease	20	(12.7)	11	(14.3)	9	(11.3)	0.568
3-vessel-disease	63	(40.1)	27	(35.1)	36	(45.0)	0.204
Percutaneous coronary intervention	76	(48.4)	30	(39.0)	46	(57.5)	**0.025**
**Classification of CS**, *n* (%)							
Stage A	0	(0.0)	0	(0.0)	0	(0.0)	**0.001**
Stage B	3	(1.9)	3	(3.9)	0	(0.0)	
Stage C	59	(37.6)	40	(51.9)	19	(23.8)	
Stage D	16	(10.2)	9	(11.7)	7	(8.8)	
Stage E	79	(50.3)	25	(32.5)	54	(67.5)	
**Transthoracic echocardiography on day 1**							
LVEF > 55%, (*n*, %)	15	(9.6)	9	(11.7)	6	(7.5)	
LVEF 54–41%, (*n*, %)	18	(11.5)	13	(16.9)	5	(6.3)	
LVEF 40–30%, (*n*, %)	36	(22.9)	22	(28.9)	14	(17.4)	**0.021**
LVEF < 30%, (*n*, %)	79	(50.3)	29	(37.7)	50	(62.5)	
LVEF not documented, (*n*, %)	9	(5.7)	4	(5.2)	5	(6.3)	
VCI, cm (median (IQR))	2.0	(1.6–2.3)	1.9	(1.5–2.4)	2.0	(1.6–2.3)	0.764
TAPSE, mm (median (IQR))	15	(11–18)	17	(11–20)	15	(11–17)	0.300
**Cardiopulmonary resuscitation**							
OHCA, *n* (%)	57	(36.3)	20	(26.0)	37	(46.3)	**0.001**
IHCA, *n* (%)	22	(14.0)	5	(6.5)	17	(21.3)	
Shockable rhythm, *n* (%)	112	(72.3)	59	(77.6)	53	(67.1)	0.143
Non-shockable rhythm, *n* (%)	43	(27.7)	17	(22.4)	26	(32.9)	
ROSC, min (median IQR)	13	(8–20)	10	(5–17)	14	(10–29)	0.082
**Respiratory status**							
Mechanical ventilation, *n* (%)	94	(40.1)	36	(46.8)	58	(72.5)	**0.001**
Duration of mechanical ventilation, days, (mean (IQR))	2	(1–6)	1	(0–6)	2	(1–6)	**0.018**
PaO_2_/FiO_2_ ratio, (median (IQR))	227	(137–336)	200	(140–334)	242	(129–354)	0.557
PaO_2_, mmHg (median (IQR))	108	(78–166)	101	(77–141)	112	(79–187)	0.138
**Pharmacotherapies**							
Norepinephrine dose on admission, µg/kg/min (median (IQR))	0.1	(0.0–0.3)	0.1	(0.0–0.2)	0.2	(0.1–0.6)	**0.001**
Dobutamine, *n* (%)	48	(30.6)	16	(20.8)	32	(40.0)	**0.009**
Levosimendan, *n* (%)	41	(26.1)	16	(20.8)	25	(31.3)	0.135
Heparin, *n* (%)	91	(58.0)	38	(49.4)	53	(66.3)	**0.032**
Bivalirudin, *n* (%)	8	(5.1)	2	(2.6)	6	(7.5)	0.277
P2Y12-inhibitor, *n* (%)	79	(50.3)	36	(46.8)	43	(53.8)	0.381
**Mechanical circulatory assist device**, *n* (%)	17	(10.8)	2	(2.6)	15	(18.8)	**0.001**
VA-ECMO	12	(7.6)	1	(1.3)	11	(13.8)	**0.003**
Impella	3	(1.9)	1	(1.3)	2	(2.5)	1.000
ECMELLA	2	(1.3)	0	(0.0)	2	(2.5)	0.497
**Renal replacement therapy**, *n* (%)	52	(33.1)	15	(19.5)	37	(46.3)	**0.001**
**Blood transfusion**, *n* (%)							
Red blood cells	50	(31.8)	24	(31.1)	26	(32.5)	0.858
Platelets	3	(1.9)	0	(0.0)	3	(3.8)	0.245
**Baseline laboratory values** (median (IQR))							
pH	7.29	(7.20–7.37)	7.30	(7.23–7.37)	7.29	(7.19–7.38)	0.635
Lactate (mmol/L)	3.4	(1.8–6.6)	2.6	(1.6–4.4)	4.6	(2.4–10.2)	**0.001**
Sodium (mmol/L)	138	(135–141)	138	(135–141)	138	(135–142)	0.588
Potassium (mmol/L)	4.3	(3.8–4.9)	4.2	(3.7–4.8)	4.4	(3.8–5.0)	0.366
Creatinine (mg/dL)	1.48	(1.14–2.39)	1.30	(1.05–1.96)	1.61	(1.32–2.80)	**0.008**
Hemoglobin (g/dL)	12.3	(10.2–13.9)	12.4	(10.1–14.2)	12.2	(10.2–13.8)	0.462
WBC (106/mL)	14.75	(10.65–18.96)	13.31	(9.54–17.64)	15.66	(12.63–20.92)	**0.003**
Platelets (106/mL)	232	(178–277)	226	(170–304)	233	(180–267)	0.725
INR	1.18	(1.08–1.43)	1.15	(1.06–1.38)	1.22	(1.10–1.47)	**0.023**
D-dimer (mg/L)	10.59	(4.14–32.00)	5.21	(2.50–14.30)	19.09	(8.20–32.00)	**0.001**
AST (U/L)	129	(45–312)	109	(38–214)	169	(59–516)	**0.016**
ALT (U/L)	74	(31–194)	53	(29–122)	98	(35–319)	**0.009**
Bilirubin (mg/dL)	0.63	(0.43–0.99)	0.60	(0.41–0.99)	0.65	(0.46–1.00)	0.395
Troponin I (µg/L)	1.118	(0.204–7.992)	0.456	(0.144–3.440)	2.861	(0.443–19.943)	**0.002**
NT-pro BNP (pg/mL)	7839	(1057–15,349)	5702	(480–14,973)	11226	(1477–21,645)	0.206
Procalcitonin (ng/mL)	0.28	(0.13–0.92)	0.31	(0.07–0.80)	0.28	(0.19–1.23)	0.559
CRP (mg/L)	13	(4–43)	11	(4–52)	20	(4–38)	0.977
**Primary endpoint**							
All-cause mortality at 30 days, *n* (%)	80	(51.0)	0	(0.0)	80	(100.0)	**0.001**
**Follow-up data**, *n* (%)							
ICU time, days (median (IQR))	4	(2–8)	4	(3–9)	3	(2–7)	**0.004**
Death ICU, *n* (%)	81	(51.6)	4	(5.2)	77	(96.3)	**0.001**

ALT, alanine aminotransferase; AST, aspartate aminotransferase; CABG, coronary artery bypass grafting; CAD, coronary artery disease; CRP, C-reactive protein; ECMELLA, Impella plus veno-arterial extracorporeal membrane oxygenation; ICU, intensive care unit; IHCA, in-hospital cardiac arrest; INR, international normalized ratio; IQR, interquartile range; NT-pro BNP, aminoterminal pro-B-type natriuretic peptide; OHCA, out-of-hospital cardiac arrest; PCI, percutaneous coronary intervention; RCA; ROSC, return of spontaneous circulation; TAPSE, tricuspid annular plane systolic excursion; VA-ECMO, veno-arterial extracorporeal membrane oxygenation; VCI, vena cava inferior; WBC, white blood cells; LVEF, left ventricular ejection fraction. Level of significance *p* < 0.05. Bold type indicates statistical significance.

**Table 3:** Univariate correlations of the AST/ALT ratio and bilirubin with laboratory and clinical parameters in all patients on day 1.

	AST/ALT Ratio		Bilirubin	
	r	*p*-Value	r	*p*-Value
**Day 1**				
Age	−0.084	0.296	0.077	0.361
Platelet count (10^6^/mL)	0.006	0.941	−0.254	**0.002**
Albumin (g/L)	−0.011	0.900	−0.164	0.058
AST/ALT ratio	-	-	0.164	0.052
Bilirubin (mg/dL)	0.164	0.052	-	-
CRP (mg/L)	0.008	0.921	0.276	**0.001**
Procalcitonin (ng/mL)	0.225	0.092	0.412	**0.002**
cTNI (µg/L)	0.366	**0.001**	0.138	0.130
NT-pro BNP (pg/mL)	0.191	0.096	0.338	**0.003**
LVEF	0.086	0.297	0.254	**0.003**
TAPSE	0.016	0.907	−0.227	0.117

ALT, alanine aminotransferase; AST, aspartate aminotransferase; CRP, C-reactive protein; cTNI, cardiac troponin I; LVEF, left ventricular ejection fraction; NT-pro BNP, N-terminal pro-B-type natriuretic peptide; TAPSE, tricuspid annular plane systolic excursion. Level of significance *p* < 0.05. Bold type indicates statistical significance.

**Table 4:** Prognostic performance of the AST/ALT ratio and bilirubin at days 1, 2, 3, 4 and 8 were analyzed as the area under the curve (95% CI).

	AST/ALT Ratio	Bilirubin	*p*-Value
**Day 1**	0.497 (0.406–0.588) *p* = 0.944	0.541 (0.446–0.637) *p* = 0.395	0.511
**Day 2**	0.539 (0.428–0.649) *p* = 0.491	0.547 (0.440–0.654) *p* = 0.388	0.919
**Day 3**	0.601 (0.475–0.727) *p* = 0.112	0.487 (0.362–0.613) *p* = 0.843	0.200
**Day 4**	**0.692** **(0.565–0.819)** ***p* = 0.008**	0.491 (0.351–0.631) *p* = 0.901	**0.042**
**Day 8**	**0.756** **(0.596–0.917)** ***p* = 0.012**	0.617 (0.427–0.807) *p* = 0.248	0.306

ALT, alanine aminotransferase; AST, aspartate aminotransferase. Level of significance *p* < 0.05. Bold type indicates statistical significance.

**Table 5:** Uni- and multivariable Cox regression analyses on day 4 within the entire study cohort with regard to 30-day all-cause mortality.

Variables	Univariable			Multivariable		
	HR	95% CI	*p*-Value	HR	95% CI	*p*-Value
Age	1.012	0.994–1.030	0.192	0.991	0.954–1.028	0.618
Sex	1.078	0.688–1.691	0.742	1.264	0.522–3.061	0.603
BMI (kg/m^2^)	1.008	0.965–1.053	0.714	1.048	0.946–1.162	0.368
Mechanical circulatory assist device	2.788	1.574–4.937	**0.001**	2.825	0.685–11.644	0.151
Renal replacement therapy	2.145	1.378–3.339	**0.001**	0.797	0.277–2.294	0.674
Creatinine (mg/dL)	1.307	0.964–1.774	0.085	1.286	0.864–1.914	0.215
Lactate (mmol/L)	1.135	1.020–1.262	**0.020**	1.410	1.100–1.809	**0.007**
Bilirubin > 0.69 (mg/dL)	1.159	0.552–2.432	0.696	0.606	0.217–1.690	0.338
AST/ALT ratio > 1.34	2.826	1.227–6.510	**0.015**	2.830	1.054–7.601	**0.039**

BMI, body mass index. Level of significance *p* < 0.05. Bold type indicates statistical significance.

## Data Availability

The datasets used and/or analyzed during the current study are available from the corresponding author upon reasonable request.

## Supplementary Materials

The following supporting information can be downloaded at: https://www.mdpi.com/article/10.3390/jcm12165275/s1, Table S1: Univariate correlations of the AST/ALT ratio and bilirubin with laboratory and clinical parameters in all patients on days 2, 3, 4 and 8.
