# Supplementary material for: Prognostic Value of the AST/ALT Ratio versus Bilirubin in Patients with Cardiogenic Shock
Source: J Clin Med. 2023 Aug 14;12(16):5275. doi: 10.3390/jcm12165275 (PMC10455678; doi:10.3390/jcm12165275)
Supplement: Supplementary file 1 [file jcm-12-05275-s001.zip › jcm-2517080-supplementary.pdf]

Supplemental Table S1. Univariate correlations of the AST/ALT ratio and bilirubin with laboratory and clinical parameters in all patients on day 2,3,4 and day 8.

	AST/ALT ratio		Bilirubin	
**Day 2**				
	**r**	**p value**	**r**	**p value**
Age	-0.087	0.368	-0.113	0.232
Platelet count (10^6/ml)	0.037	0.701	-0.311	**0.001**
Albumin (g/l)	0.019	0.843	-0.068	0.474
AST/ALT ratio	-	-	0.239	**0.013**
Bilirubin (mg/dl)	0.239	**0.013**	-	-
CRP (mg/l)	0.069	0.482	0.138	0.146
Procalcitonin (ng/ml)	0.470	**0.013**	0.198	0.313
cTNI (µg/l)	0.564	**0.001**	0.087	0.438
NT-pro BNP (pg/ml)	-0.162	0.332	-0.171	0.286
**Day 3**				
Age	-0.057	0.598	-0.106	0.328
Platelet count (10^6/ml)	-0.040	0.715	-0.493	**0.001**
Albumin (g/l)	0.016	0.883	0.109	0.325
AST/ALT ratio	-	-	0.180	0.101
Bilirubin (mg/dl)	0.180	0.101	-	-
CRP (mg/l)	0.274	**0.011**	0.062	0.576
Procalcitonin (ng/ml)	0.229	0.224	0.100	0.599
cTNI (µg/l)	0.642	**0.001**	-0.197	0.179
NT-pro BNP (pg/ml)	0.167	0.414	-0.066	0.755
**Day 4**				
Age	-0.007	0.954	-0.073	0.556
Platelet count (10^6/ml)	-0.120	0.334	-0.539	**0.001**
Albumin (g/l)	-0.052	0.682	-0.151	0.229
AST/ALT ratio	-	-	0.263	**0.034**
Bilirubin (mg/dl)	0.263	**0.034**	-	-
CRP (mg/l)	0.440	**0.001**	0.226	0.066
Procalcitonin (ng/ml)	0.293	0.176	0.276	0.192
cTNI (µg/l)	0.369	0.053	-0.017	0.928
NT-pro BNP (pg/ml)	-0.491	0.125	-0.264	0.433
**Day 8**				
Age	0.030	0.858	-0.086	0.601
Platelet count (10^6/ml)	-0.342	**0.039**	-0.549	**0.001**
Albumin (g/l)	-0.364	**0.029**	-0.145	0.393
AST/ALT ratio	-	-	0.336	**0.039**
Bilirubin (mg/dl)	0.336	**0.039**	-	-
CRP (mg/l)	0.345	**0.034**	0.497	**0.001**
Procalcitonin (ng/ml)	0.244	0.362	0.759	**0.001**
cTNI (µg/l)	0.400	0.600	0.400	0.600
NT-pro BNP (pg/ml)	0.573	0.051	0.615	0.033

ALT, alanine aminotransferase; AST, aspartate aminotransferase; CRP, C-reactive protein; cTNI, cardiac troponin I; NT-pro BNP, N-terminal pro-B-type natriuretic peptide.
Level of significance $p<0.05$. Bold type indicates statistical significance.

.
